# Single Institution Experience of Stereotactic Body Radiation Therapy in Non-small Cell Lung Cancer: Comparison of Two Dose Regimes and a Perspective on Ideal Dose Regimens

**DOI:** 10.7759/cureus.18862

**Published:** 2021-10-18

**Authors:** Mary R Nittala, William N Duggar, Eswar Mundra, Satya Packianathan, Maria L Smith, William C Woods, Jeremy Otts, Rahul Bhandari, Robert Allbright, Pierre E De Delva, Jacob R Moremen, Claus Chunli Yang, Srinivasan Vijayakumar

**Affiliations:** 1 Radiation Oncology, University of Mississippi Medical Center, Jackson, USA; 2 Radiation Oncology, G.V. (Sonny) Montgomery VA Medical Center, Jackson, USA; 3 Surgery, University of Mississippi Medical Center, Jackson, USA

**Keywords:** overall survival (os), stereotactic body radiation therapy, lung cancer, squamous cell carcinoma, adenocarcinoma

## Abstract

Introduction

Stereotactic body radiation therapy (SBRT) is an effective treatment for early-stage non-small cell lung cancer (NSCLC) patients who are either medically inoperable or who decline surgery. SBRT improves tumor control and overall survival (OS) in medically inoperable, early-stage, NSCLC patients. In this study, we investigated the effectiveness of two different SBRT doses commonly used and present our institutional experience.

Purpose

To determine the clinical outcomes between two treatment regiments (50 Gray [Gy] *vs.* 55 Gy in five fractions) among Stage I NSCLC patients treated with SBRT at a state academic medical center.

Methods

We performed a retrospective analysis of 114 patients with Stage I (T1-2 N0 M0) NSCLC treated at a state academic medical center between October 2009 and April 2019. Survival analyses with treatment regimens of 50 Gy and 55 Gy in five fractions were conducted to detect any improvement in outcomes associated with the higher dose. The primary endpoints of this study included OS, local control (LC), and disease-free survival (DFS). Log-rank test and the Kaplan-Meier method were used to analyze the survival curves of the two treatment doses. The SPSS v.24.0 (IBM Corp., Armonk, NY, USA) was used for statistical analyses.

Results

The 114 early-stage NSCLC patients (median age, 68 years; range 12 to 87 years) had a median follow-up of 25 months (range two to 86 months). The number of males (n = 72; 63.2 %) exceeded the number of females (n = 42; 36.8 %). The majority of patients in this study were Caucasians (n = 68; 59.6 %) and 46 patients were African Americans (40.4 %). Two-thirds of the patients (n = 76; 66.7 %) were treated with 50 Gy in five fractions, and 38 patients (33.3 %) with 55 Gy in five fractions. The one-, two-, and three-year OS and DFS rates were improved in the patients treated with 55 Gy [OS, 81.7 % *vs.* 72.8 %; 81.7 % *vs.* 58.9 %; 81.7 % *vs*. 46.7 % (p = 0.049)], [DFS, 69.7 % *vs.* 69.7 %; 61.9 % *vs.* 55.7 %; 61.9 % *vs.* 52.0 % (p = 0.842)], compared to those treated with 50 Gy. Adenocarcinoma was the most common histology in both groups (51.3 % and 68.4 %). Failure rates were elevated for the 50 Gy regimen [39 (34.2 %) *vs*. 12 (8.5 %)]. Three year control rates were (66.3 % *vs*. 96.6 %; p = 0.002) local control; (63.3 % *vs.* 94.4 %; p = 0.000) regional control; and (65.7 % *vs.* 97.1 %; p = 0.000) distant control, compared to those treated with 55 Gy.

Conclusion

Early-stage NSCLC patients treated with SBRT 55 Gy in five fractions did better in terms of local control, overall survival, and disease-free survival rates compared to the 50 Gy in five fractions group.

## Introduction

Lung cancer is on the rise globally and is the most common cause of cancer death [1]. In 2020, Global Cancer Incidence, Mortality and Prevalence (GLOBOCAN) estimated 2.20 million new cases and 1.79 million deaths [2,3], making it the most common cancer and cause of cancer death [4]. Lung cancer has been the leading cause of cancer-related deaths for many years in the United States [incidence (235,000) and mortality (131,000)] per the 2021 estimates [3]. About a quarter of patients diagnosed with NSCLC present with stage I disease; they are the most curable cohort of the NSCLC population [2,3]. For stages I, and II, without medical comorbidities, surgery is the treatment of choice with five-year survival rates ranging from 60 to 80% for stage I, and 40 to 50% for stage II, respectively [5]. Stereotactic body radiation therapy (SBRT) is a newer radiotherapy treatment modality that has been used in the treatment of medically inoperable early-stage (NSCLC) patients. It affords excellent survival results, giving a high quality of life and a high local control (LC) rate of 70 to 90% [6-9]. Moreover, SBRT is cost-effective and provides a stable, global quality of life during the first year after treatment [10,11]. Four to 15 fraction regimens are commonly used in this setting and are usually preferable to a conventionally fractionated approach [12]. Most of the guidelines from studies performed suggest doses of 48 to 60 Gray (Gy) in three to eight fractions, delivered over about three weeks [13]. A combined analysis of two randomized studies of SBRT versus surgery for stage I NSCLC patients showed improved survival rates with SBRT [14]. A higher biologically effective dose (BED) delivered during a short period of time has been associated with higher local control rates of NSCLC [15].

Over the previous two decades, hypo-fractionated high-dose SBRT has been used for treating stage I NSCLC patients. SBRT was developed to deliver a high BED to T1-2 lesions while controlling doses delivered to normal tissues. Stereotactic targeting of these tumors in combination with specialized 3-D conformal therapy using techniques such as dynamic conformal arcs allows the use of highly focused radiation from various angles and even different planes to cover a “moving” target - the tumor in a breathing lung. Many SBRT studies have previously reported the variability in outcomes with different clinical target volume (CTV) and planning target volume (PTV) margins, dose prescription techniques, treatment planning parameters and, dose calculation algorithms, and use of 4-D treatment planning CT scans [16]. Many prospective, retrospective, and population-based studies which used hypo-fractionated radiation therapy schema have also reported increased LC and OS rates compared to surgery [14,15]. In this study, we present our institutional SBRT experience using two treatment regimens: 50 Gy *vs.* 55 Gy in five fractions for stage I NSCLC patients and determine their clinical outcomes.

## Materials and methods

Study design and participants

This retrospective analysis of 114 early-stage NSCLC patients treated with SBRT between 2009 and 2019 was approved by the University of Mississippi Medical Center Institutional Review Board (IRB #2018-0086). The treatment regimen and patient numbers were as follows: 95.6 % patients treated to 50 Gy and 4.4 % to 55 Gy between 2009 to 2016. Additionally, between January 2017 and 2019, 23.9 % were treated to 50 Gy and 76.1 % treated to 55 Gy.

A browser-based database tool, Research Electronic Data Capture (REDCap), was used to extract and store the patients' information in password-protected computers. Diagnoses and clinical staging of lung being fundamental to patient therapy, computed tomography (CT) of the chest and abdomen, magnetic resonance imaging (MRI) or CT of the brain, PET/CT, bone scans, pulmonary function tests, and laboratory tests were routinely performed per established National Comprehensive Cancer Network (NCCN) guidelines.

Data collection

All the patients’ details regarding demographics, disease presentation, disease staging, treatments, and complications were documented in REDCap at the time of their enrollment into this retrospective analysis. Before the information was extracted, all patient identifiers were stripped. All the 114 patients included in this retrospective study were categorized as early-stage NSCLC, according to the staging manual of the American Joint Committee on Cancer (AJCC), 8th edition. The following patient characteristics were collected: date of diagnosis, gender, age, ethnicity, body mass index (BMI), insurance status, tumor grade, tumor location, treatment dose, and survival in months. All the data were collected, checked, analyzed, and interpreted by the postdoctoral - research fellow (MN) and verified by co-authors.

Treatment details

CT Simulation

**Figure 1:**
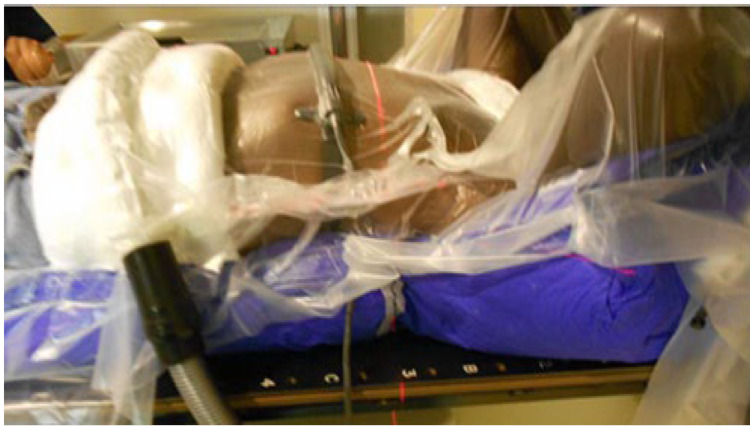
Patient immobilized for 4D simulation utilizing a Body-Fix bag with evacuated plastic and a bellows belt for respiratory cycle capture

Prior to treatment planning, all patients underwent a 4-D CT simulation utilizing either a Siemens SomatomS simulator (Siemens Medical Solutions Inc., Malvern, PA, USA) with an Anzai pressure system (Anzai Medical Co. Ltd., Tokyo, Japan) or a Philips Big Bore Simulator (Philips N.V., Cambridge, MA, USA) with a respiratory bellows system. Patients were immobilized using an Elekta BodyFix system (Elekta Inc., Stockholm, Sweden) with pressure set to 100 PSI. A 4-D CT was acquired in addition to a planning CT which extended from the patient’s chin through the total lung volume into the mid-abdomen, based on their respiratory cycle (Figure 1).

Following the CT, an initial isocenter was defined using the respective CT scanner’s virtual simulation software. Images were then exported to the treatment planning system and setup marks were documented on the patient’s skin with permanent ink and/or tattoos. Post-simulation, each patient and their immobilization device was transported to the treatment room where a “dry-run” was performed to identify potential collision positions during treatment delivery and to finalize the isocenter position for optimal gantry/couch clearance.

Treatment Planning

**Figure 2:**
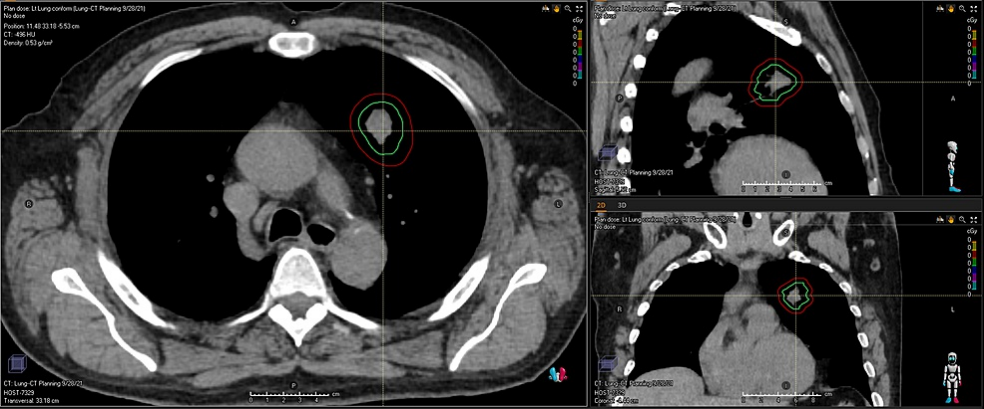
A three-view representation of the contours ITV (green) and PTV (red) for use in SBRT Lung. ITV: internal target volume, PTV: planning target volume, SBRT: stereotactic body radiation therapy

Upon import into the treatment planning system, image fusion was performed utilizing the MIMs software and an aligned version of the PET-CT at the area of interest was imported into the planning system (either Philips Pinnacle (Philips Inc., NV, USA) or Raysearch Raystation (Raysearch Labs., Stockholm, Sweden)). Targets and normal tissues were then defined on the planning CT per the guidelines established by Radiation Therapy Oncology Group (RTOG) 0813 with the addition of rib contours. The gross tumor volume (GTV) was defined based on its extent on the CT and the aligned PET. The 4-D-CT was used to generate an internal target volume (ITV) of the GTV. Subsequently, a planning target volume (PTV) was created using a 5 mm expansion of the ITV (Figure 2).

**Figure 3:**
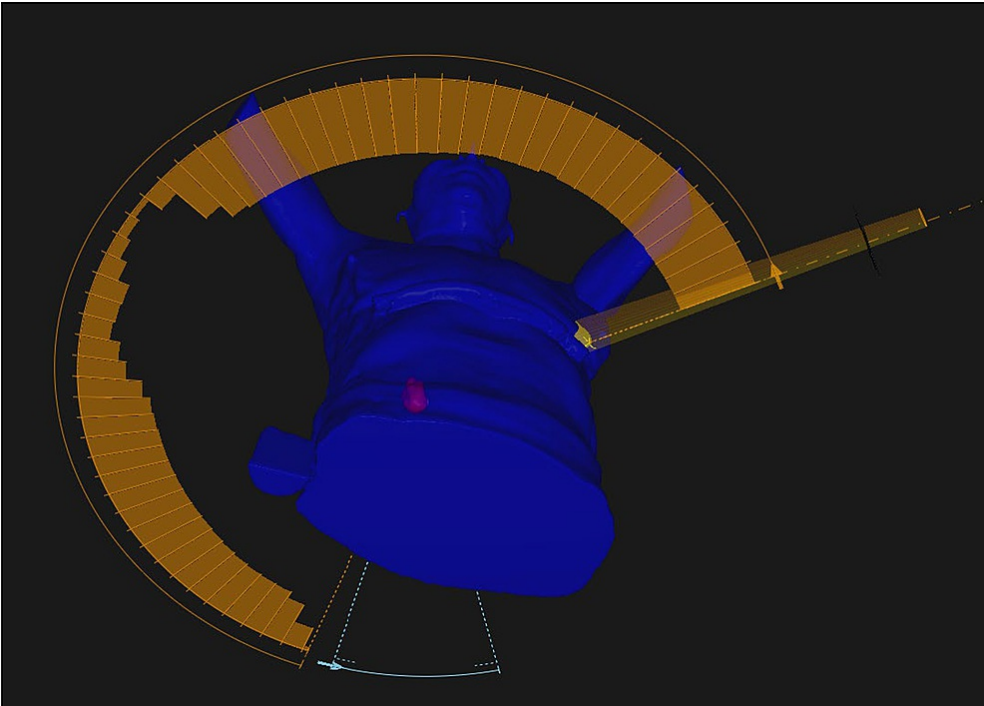
3D rendering of conformal arcs SBRT Treatment Plan 3D: three- dimensional, SBRT: stereotactic body radiation therapy

**Figure 4:**
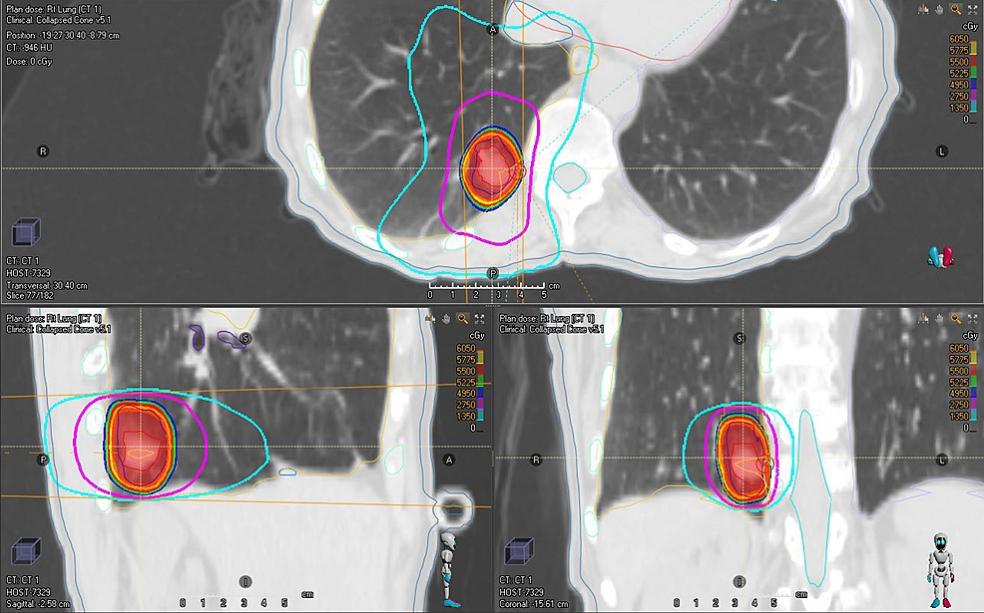
typical conformal arc SBRT lung dose distribution SBRT: stereotactic body radiation therapy

Following contouring, treatment planning usually involved the definition of two to three conformal arcs, weighted so as to optimally meet conformity goals in addition to normal tissue constraints while covering at least 95 % of the PTV with prescription dose and 99 % of the PTV with 90 % of the prescription dose. Beam energy most commonly used was 6 MV, but eventually, 6 MV flattening filter free (FFF) became the preferred clinical choice once it became available. The prescription isodose line was selected to optimize PTV coverage and conformity and was only permitted to lie within a range of 60 - 90 % per RTOG 0813. The beam margin around the PTV was optimized to prevent dose spillage outside of the PTV because of higher density regions and loss of PTV coverage secondary to lower density regions. Conformity goals were defined based on RTOG 0813 and normal tissue goals were compliant with RTOG 0813 and TG-101 recommendations (Figure 3, 4). 

**Figure 5:**
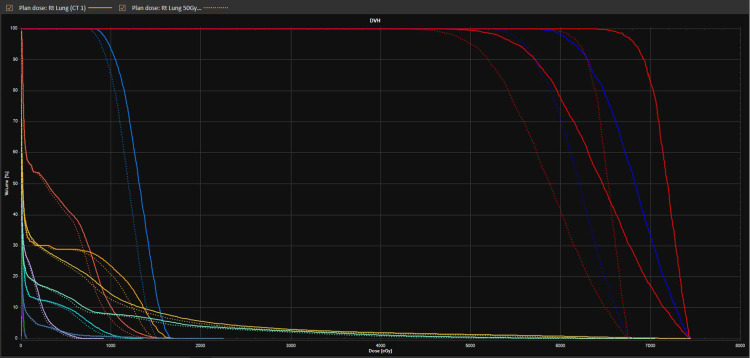
DVH comparison of the same treatment plan design, but at different prescriptions: 55 Gy (solid) vs 50 Gy (dashed) DVH: dose volume histogram

Once planning was completed (Figure 5), all patients underwent pre-treatment peer review by a multi-disciplinary conference [17].

Treatment Delivery

**Figure 6:**
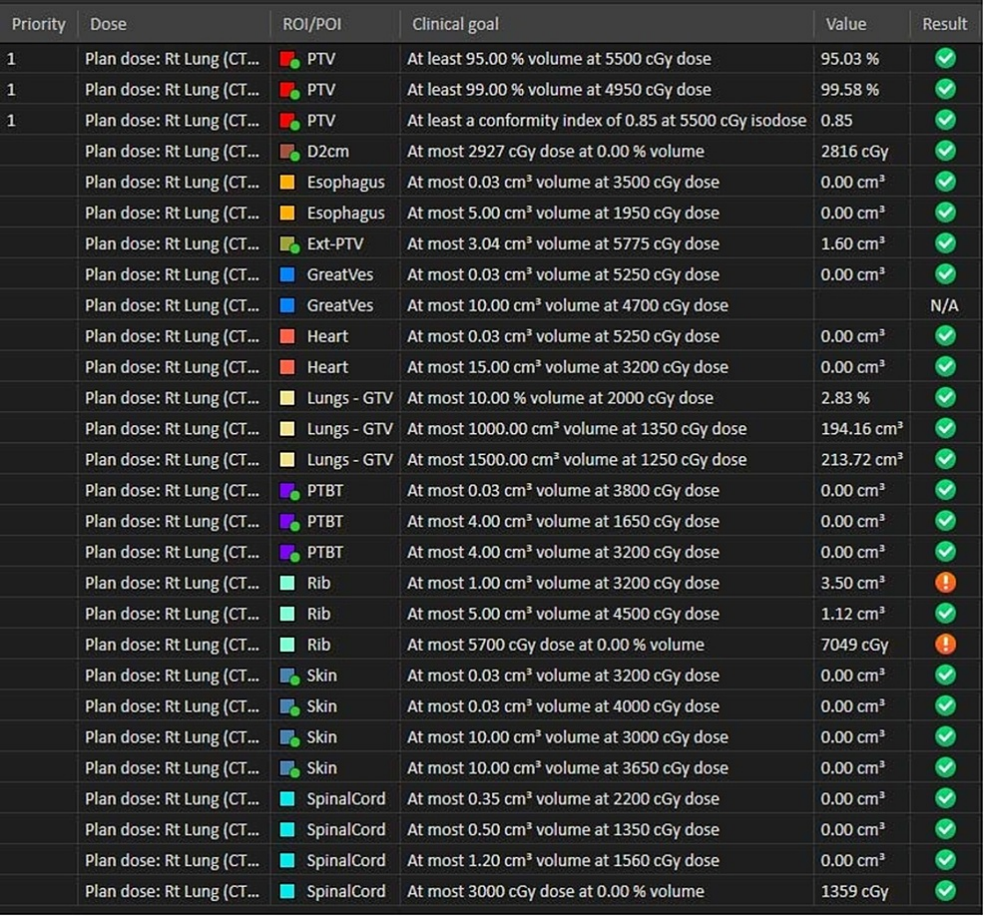
Departmental SBRT lung 5-fraction Dose Constraint Scorecard SBRT: stereotactic body radiation therapy, PTV: planning target volume, GTV: gross tumor volume, PTBT: proximal tracheobronchial tree.

Based on American College of Radiology (ACR) guidelines, a radiation oncologist and a physicist were present for the entirety of every treatment fraction. Patients were positioned in their custom immobilization device based on the marks from simulations before the treatment plan prescribed patient shifts were applied. X-ray volumetric imaging (XVI) was performed (either an Elekta SynergyS (Elekta Inc., Stockholm, Sweden) or an Elekta VersaHD (Elekta Inc., Stockholm, Sweden)). If the initial shifts were greater than 0.5 cm in any direction, the shifts were applied and the XVI imaging was repeated. The final shifts were reviewed by therapists, a physician, and a physicist (Figure 6). 

Once all were in agreement, treatment was initiated, with the patient under observation to detect any movement (coughing, etc). Treatment was typically delivered every other day to minimize toxicity. Upon completing the final fraction, follow-up and imaging appointments were made.

Definitions

Disease-free survival (DFS) is defined as the number of days from date of initial diagnosis through treatment to first recurrence of disease or cancer. Overall survival (OS) is defined by the number of days from the date of initial diagnosis until the date of death/the last contact. The censored cases were defined as the patients still alive at the time of the last follow-up. Local control (LC), defined as the prevention of cancer growth at the site of treatment. Treatment outcomes can be described as the extent of disease control locally, degree of disease control regionally and the prevention of disease recurrence at distant sites following their curative treatment.

Statistical analysis

The Pearson’s chi-square test was used to identify the relationship between the two categorical variables and the respective p-values were recorded. The Kaplan-Meier method was used to estimate the OS and DFS rates and the statistical significance between the survival curves of the two groups. The one-, two-, and three-year OS and DFS rates by treatment group were estimated from the cumulative proportion surviving at the particular time (survival table). All P values ≤ 0.05 were considered statistically significant. Data were analyzed using SPSS 24.0 software (IBM Corp., Armonk, NY, USA).

## Results

Patient characteristics

**Figure 7:**
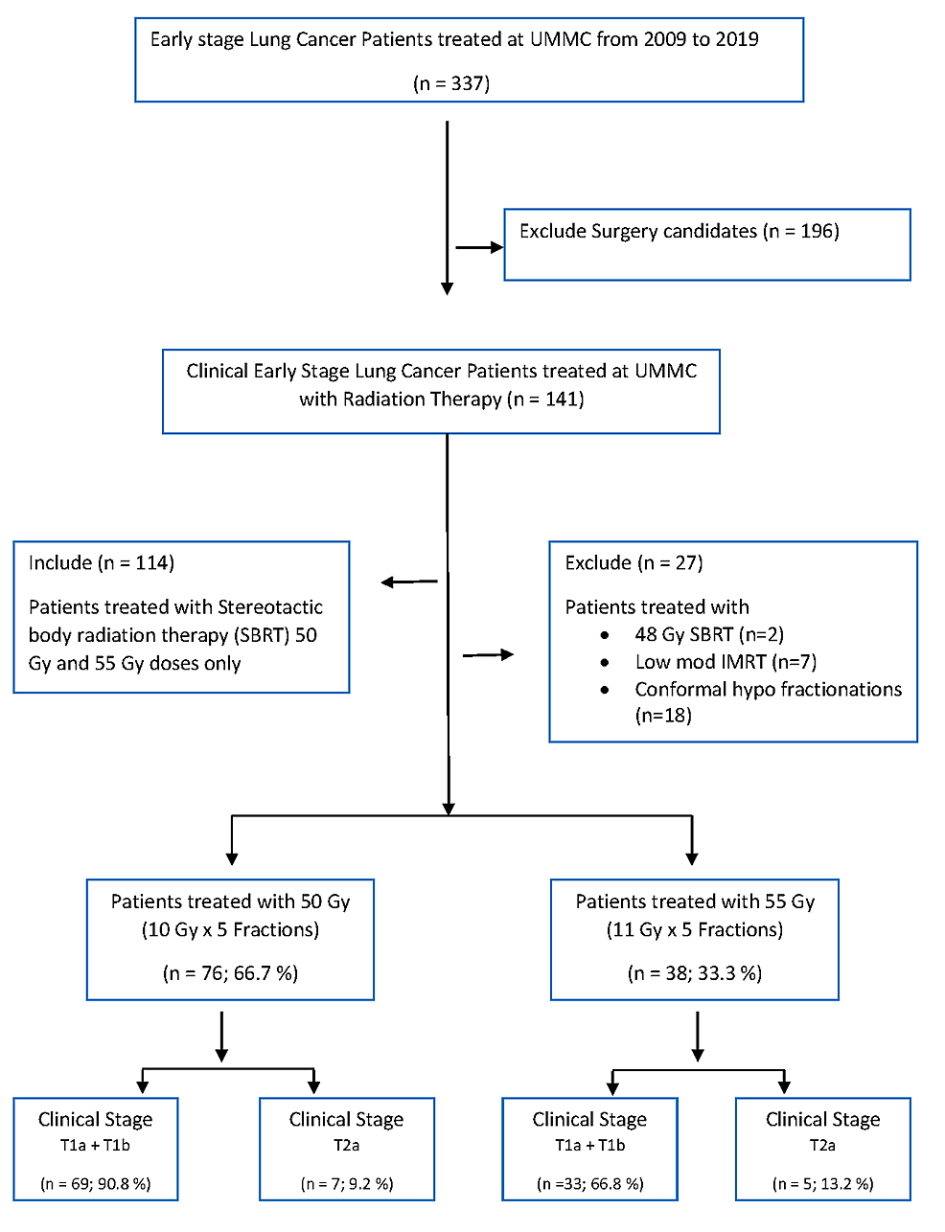
Flow chart for lung cancer patients cohort selection UMMC: University of Mississippi Medical Center, SBRT: Stereotactic body radiation therapy, IMRT: intensity modulated radiation therapy, Gy: gray, n: number, %: percentage.

A total of 114 early-stage NSCLC patients were treated between 2009 and 2019. These cases were considered for SBRT because either they were medically inoperable or because they refused surgery. Out of 114 patients, 76 (66.7 %) were treated with 50 Gy and 38 (33.3 %) with 55 Gy. We have previously reported our outcomes based primarily on a dose of 50 Gy in five fractions. Dissatisfied with our reported outcomes, the department policy was modified to deliver 55 Gy in five fractions, if the treatment plan met the normal tissue constraint standards of the department. All patients’ treatment targets and normal tissue structures, treatment plans in respect of target coverage and normal tissue constraints were reviewed and approved in a multidisciplinary pre-treatment conference prior to initiating treatment [17]. Seventy-six of our patients received 50 Gy during the first time period up to December 2016 while 38 patients received 55 Gy in the sequential second time frame after the change in the department policy (Figure 7).

The baseline characteristics of the study population are shown in Table 1. The median age of the study population was 68 years (range 12 to 87 years). A greater number of the male patients were treated with 50 Gy *vs.* 55 Gy (68.4 %* vs.* 52.6 %; p = 0.009). Overall, the patients treated with 50 Gy had higher BMI levels compared to the ones treated with 55 Gy (31.5 % *vs.* 14 %; p = 0.375). Like BMI, socio-economic factors, insurance status, distance traveled for treatment, smoking, and alcohol use history did not present any statistically significant differences between the two treatment groups.

**Table 1:** Lung cancer SBRT patients characteristics SBRT: stereotactic body radiation therapy, n: number, %: percentage.

	50 Gy	55 Gy	p- value
Patients in review , n = 114	76	38	
Years			
2009- 2016	65 (85.5%)	3 (7.9%)	0.000
2017- 2019	11 (23.9%)	35 (92.1%)	
Gender, n (%)			
Male	52 (68.4%)	20 (52.6%)	0.099
Female	24 (31.6%)	18 (47.4%)	
Median age (range), years	68 years; (range: 12 to 87)		
Ethnicity, n (%)			
Caucasians	48 (63.2%)	20 (52.6%)	0.280
African American	28 (36.8%)	18 (47.4%)	
Body Mass Index (kg/m^2^), n (%)			
Underweight (< 18.5)	14 (18.4%)	4 (10.5%)	0.375
Normal (18.5 - 24.9)	26 (34.2%)	18 (47.4%)	
Overweight (25.0 - 29.9)	25 (32.9%)	9 (23.7%)	
Obese (> 30.0)	11 (14.5%)	7 (18.4%)	
Household Income $, n (%)			
≤ $30,000	25 (32.9%)	13 (34.2%)	0.888
>$ 30,000	51 (67.1%)	25 (65.8%)	
Insurance, n (%)			
Medicaid/VA	43 (56.6%)	16 (42.1%)	0.360
Medicare	28 (36.8%)	17 (44.7%)	
Private	3 (3.9%)	4 (10.5%)	
Self-Pay	2 (2.6%)	1 (2.6%)	
Distance Travelled, n (%), miles			
≤ 30 miles	25 (32.9%)	23 (60.5%)	0.018
30-75 miles	22 (28.9%)	7 (18.4%)	
> 75 miles	29 (38.2%)	8 (21.1%)	
Smoking History, n (%)			
Yes	72 (94.7%)	34 (89.5%)	0.300
No	4 (5.3%)	4 (10.5%)	
Current Smoking status, n (%)			
Yes	26 (34.2%)	16 (42.1%)	0.410
No	50 (65.8%)	22 (63.2%)	
Alcohol History, n (%)			
Yes	18 (23.7%)	15 (39.5%)	0.080
No	58 (76.3%)	23 (60.5%)	

In both the treatment groups (50 Gy and 55 Gy), the majority of patients presented with T1 lesions (89.5 %); adenocarcinoma was the most common histological type (57 %). The right upper lobe was the most commonly involved lobe (34.2 %). The median planning target volume (PTV) was 30.4 cm^3^ (range 7.2 - 166.6 cm^3^). The median gross tumor volume (GTV) size was 3.9 cm^3^ (range 0.3 - 44.3 cm^3^). Tumor characteristics of both treatment groups are further summarized in Table 2.

**Table 2:** Lung cancer SBRT tumor charcateristics SBRT: stereotactic body radiation therapy, RUL: right upper lobe, RLL: right lower lobe, RML: right middle lobe, LUL: left upper lobe, LLL: left lower lobe, Gy: gray, n: number, %: percentage

	50 Gy (n=76)	55 Gy (n=38)	p- value
T1a + T1b lesions, n (%)	69 (90.8%)	33 (86.8%)	0.517
T2a Lesions, n (%)	7 (9.2%)	5 (13.2%)	
Tumor Location, n (%)			
RUL lesions	28 (36.8%)	11 (28.9%)	0.430
RLL lesions	11 (14.5%)	9 (23.7%)	
RML lesions	6 (7.9%)	2 (5.3%)	
LUL lesions	20 (26.3%)	7 (18.4%)	
LLL lesions	11 (14.5%)	9 (23.7%)	
Pathology, n (%)			
Adenocarcinoma	39 (51.3%)	26 (68.4%)	0.208
Squamous cell carcinoma	29 (38.2%)	10 (26.3%)	
Lung Nodules	8 (10.5%)	2 (5.3%)	
Gross Tumor Volume (GTV) details			
Median GTV (range), cm^3^			
GTV < 8 cm^3^	56 (73.7%)	29 (76.3%)	0.951
GTV 8 - 16 cm^3^	13 (17.1%)	6 (15.8%)	
GTV > 16 cm^3^	7 (9.2%)	3 (7.9%)	
Planning Target Volume (PTV) details			
Median PTV (range), cm^3^			
PTV < 24 cm^3^	29 (38.2%)	12 (31.6%)	0.761
PTV 24 - 45 cm^3^	25 (32.9%)	13 (34.2%)	
PTV > 45 cm^3^	22 (28.9%)	13 (34.2%)	
Skin Toxicity, n (%)			
Grade 0	54 (71.1%)	28 (73.7%)	0.951
Grade 1	4 (5.3%)	2 (5.3%)	
Grade Unknown	18 (23.7%)	8 (21.1%)	
Lung Toxicity, n (%)			
Grade 0	56 (73.7%)	29 (76.3%)	0.951
Grade 1	2 (2.6%)	1 (2.6%)	
Grade Unknown	18 (23.7%)	8 (21.1%)	
Local Failure	11 (9.6%)	6 (5.3%)	0.853
Regional Failure	18 (15.8%)	3 (2.6%)	0.040
Distant Failure	10 (8.8%)	3 (2.6%)	0.405

The primary endpoints of this study are OS, DFS, LC, regional control (RC) , and distant metastasis (DM). The one-, two-, and three-year OS and DFS are summarized in Table 3.

**Table 3:** 1-, 2-, and 3-year overall survival and disease free survival (%) Gy: gray, %: percentage, PTV: planning target volume, SCCa: squamous cell histology

	Group	I year		2 years		3 years		P- value
		50 Gy	55 Gy	50 Gy	55 Gy	50 Gy	55 Gy	
Overall Survival	Entire cohort	72.8%	81.7%	58.9%	81.7%	46.7%	81.7%	0.049
	Tumor Stage 1	70.0%	86.1%	57.7%	86.1%	46.2%	86.1%	0.056
	Tumor Stage 2	71.4%	60.0%	71.4%	60.0%	53.6%	60.0%	0.056
	Path-Adenocarcinoma	76.3%	83.1%	59.3%	83.1%	46.1%	83.1%	0.076
	Pathology- SCCa	75.0%	77.8%	60.9%	77.8%	48.7%	77.8%	0.076
	Upper Lobe	71.8%	82.1%	59.4%	82.1%	46.9%	82.1%	0.060
	Middle Lobe	75.0%	-	75.0%	-	50.0%	-	0.060
	Lower Lobe	67.4%	86.7%	52.9%	86.7%	45.4%	86.7%	0.060
	FX Size 10 Gy *vs*. 11 Gy x 5	72.8%	81.7%	58.9%	81.7%	46.7%	81.7%	-
	PTV < 24 cm^3^	68.1%	91.7%	51.1%	91.7%	38.3%	91.7%	0.049
	PTV 24 - 45 cm^3^	74.7%	88.9%	64.0%	88.9%	52.4%	88.9%	0.049
	PTV > 45 cm^3^	77.3%	92.3%	63.6%	64.6%	50.9%	64.6%	0.049
Disease free survival	Entire cohort	69.7%	69.7%	55.7%	61.9%	52.0%	61.9%	0.842
	Tumor Stage 1	67.6%	70.1%	62.2%	61.4%	58.1%	61.4%	0.919
	Tumor Stage 2	85.7%	60.0%	17.1%	60.0%	17.1%	60.0%	0.919
	Path-Adenocarcinoma	78.3%	68.3%	56.6%	54.7%	42.4%	54.7%	0.981
	Pathology- SCCa	55.6%	70.0%	49.5%	70.0%	41.2%	70.0%	0.981
	Upper Lobe	78.0%	77.8%	61.6%	77.8%	61.6%	77.8%	0.563
	Middle Lobe	50.0%	-	50.0%	-	50.0%	-	0.563
	Lower Lobe	57.7%	62.2%	44.9%	46.7%	29.9%	46.7%	0.563
	FX Size 10 Gy *vs*. 11 Gy x 5	69.7%	69.7%	55.7%	61.9%	52.0%	61.9%	-
	PTV < 24 cm^3^	70.5%	91.7%	56.4%	91.7%	42.3%	91.7%	0.763
	PTV 24 - 45 cm^3^	57.1%	64.1%	44.4%	64.1%	44.4%	64.1%	0.763
	PTV > 45 cm^3^	81.0%	57.7%	66.2%	38.5%	66.2%	38.5%	0.763

The median follow-up for entire cohort was 25 months (range two to 86 months). The 55 Gy treatment group (with median follow-up of 20.5 months; range two to 52 months) showed significantly better one-, two-, and three-year OS rates compared to 50 Gy (with median follow-up of 29 months; range three to 86 months); (81.7 % *vs*. 72.8 %, 81.7 % *vs.* 58.9 %, and 81.7 % *vs*. 46.7 %; p = 0.049). The one-, two-, and three-year OS rates for 55 Gy treatment group with T1 lesions was also shown better OS rates compared to the 50 Gy (86.1 % *vs.* 70.0 %, 86.1 % *vs.* 57.7 %, and 86.1 % *vs.* 46.2 %; p = 0.056). Those patients with PTV < 24 cm^3 ^and treated to 55 Gy showed significantly better three-year OS rate compared to similar patients treated to 50 Gy (91.7 % *vs.* 38.3 %; p = 0.049). A similar pattern of better OS rate was documented for the 55 Gy treatment group with adenocarcinoma. However, DFS and OS were unaffected by squamous cell histology (SCCa), upper lobe location, lower lobe tumor location.

**Figure 8:**
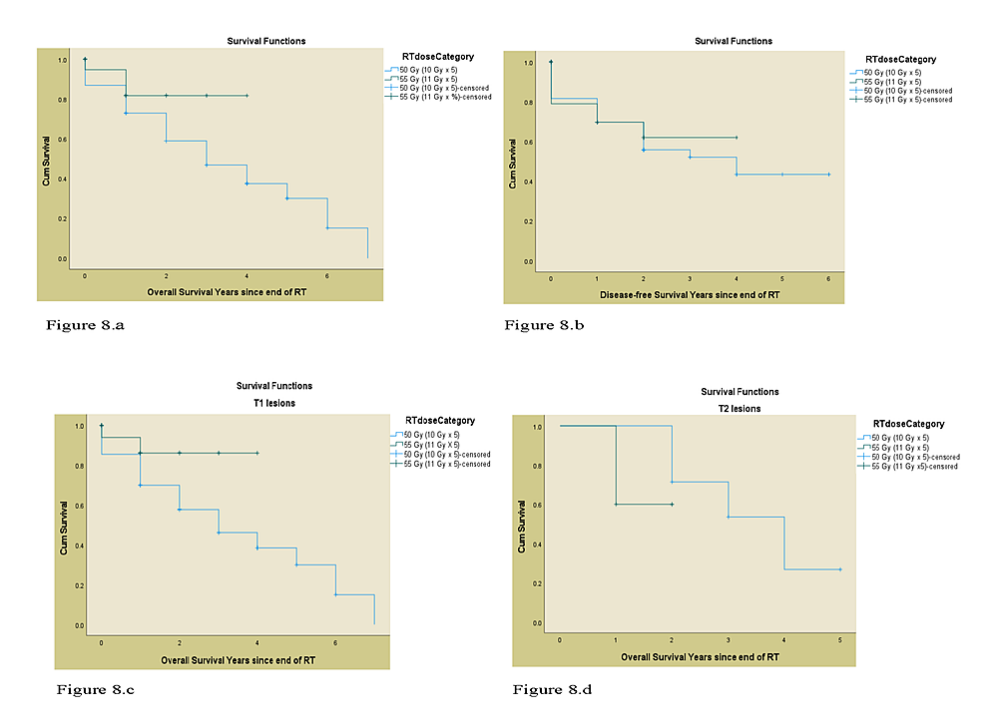
a) The Kaplan -Meier OS curve for RT dose (50 Gy Vs. 55 Gy), b) The Kaplan- Meier DFS curve for RT dose (50 Gy Vs. 55 Gy), c) The Kaplan- Meier OS curve for T1 lesions,. d) The Kaplan – Meier OS curve for T2 lesions OS: overall survival, DFS: disease free survival, RT: radiation therapy, Gy: gray, Vs.: versus

**Figure 9:**
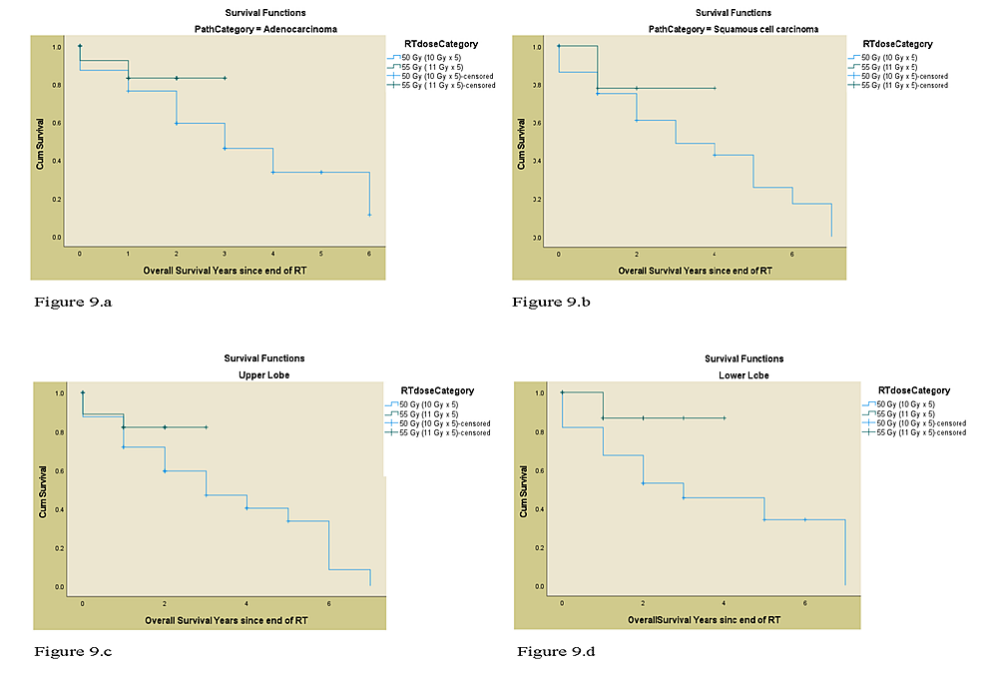
a) The Kaplan-Meier OS curve for adenocarcinoma, b) The Kaplan – Meier OS curve for squamous cell carcinoma, c) The Kaplan- Meier OS curve for upper lobe tumor location, d) The Kaplan -Meier OS curve for lower lobe tumor location OS: overall survival, RT: radiation therapy, Gy: gray.

In terms of DFS, the 55 Gy treatment group had better rates compared to 50 Gy for the entire cohort, as well as for patients with T1, T2 SCCa lesions. Tumor location was unassociated with any advantage with 55 Gy treatment (Table 3). The OS and DFS curves of both the treatment groups (55 Gy and 50 Gy) by T stage, histology, and the lobe involved are shown in Figure 8 and Figure 9, respectively.

The three-year LC, RC, and DM rates in the 55 Gy treatment were better compared to those treated with 50 Gy (p = 0.002, p = 0.000, p = 0.000) (Table 4).

**Table 4:** Kaplan - Meier survival table (%) Gy: gray, %: percentage

Total cohort (Three-year)	50 Gy	55 Gy	p-value
Local Control	66.3%	96.6%	0.002
Regional Control	63.3%	94.4%	0.000
Distant Metastasis	65.7%	97.1%	0.000

 The complications for both 50 Gy and 55 Gy are summarized in Table 5. There were no grade 4 or 5 complications. 

**Table 5:** Complications after SBRT treatment in lung cancer patients. SBRT: stereotactic body radiation therapy, Gy: gray, n: number, %: percentage

	50 Gy	55 Gy	Total	P- value
Patients in review (n)	76	38	114	
Complications				
Shortness of Breath	6 (5.3%)	1 (0.9%)	7 (6.1%)	0.628
Scarring	5 (4.4%)	3 (2.6%)	8 (7.0%)	
Rib fractures	4 (3.5%)	1 (0.9%)	5 (4.4%)	

## Discussion

SBRT has emerged as a treatment of choice for inoperable stage I NSCLC [6-9,18]. SBRT is a radiotherapy treatment technique, which compared to conventional chemo-radiation therapy, permits stereotactic delivery of a high radiation dose to a confined area around the tumor. This method is considered as an alternative to surgery for operable Stage I NSCLC patients [18]. The term “stereotactic” originally related to precise positioning in 3-D space. In this situation, this positioning, obtained by correlating the tumor target position to reliable fiducials with “already” established positions. Such fiducials define a coordinate system that can be used to target the tumor, orient the treatment planning process, and ultimately guide the therapy toward the intended location in the body [19]. Recently, as on-board X-ray imaging systems were integrated into a treatment machine hardware and software, the external fiducial marker system has been replaced by daily imaging guidance.

SBRT has been shown to provide excellent local control in the treatment of lung lesions from early-stage lung carcinoma lung metastases [12,20,21] with minimal toxicity. Severe clinical toxicity after SBRT is uncommon and occurs more frequently in the treatment of the more centrally located tumors, such as those near the trachea, primary bronchus, major blood vessels, or pericardium [12]. Similar, to chemo radiation-induced CT changes after treatment, CT findings after SBRT can also be classified into two stages: early (within six months), i.e., acute radiation pneumonitis, and late (later than six months), i.e., radiation fibrosis [22,23]. Higher radiotherapy doses have been associated with better survival in NSCLC patients treated with SBRT [24].

As the accurate delivery of targeted radiation therapy improved over time, SBRT emerged as a technique to deliver a high dose of radiation precisely utilizing a small number of fractions [25]. Initially, SBRT treatments were mainly performed at 48 - 52 Gy in four fractions [26]. The reported OS and LC at two years for NSCLC (T1N0M0) patients were 79 %, and 76.4 %, respectively with no Grade 3 or higher toxicity reported [27]. Our institution initially used 10 Gy x five fractions, which has a BED of 100 Gy. (The current fractionation of 11 Gy x five has a BED of 110 Gy).

Our entire patient cohort was separated into 10 Gy x five *vs.* 11 Gy x five fractions. Our reported OS and DFS at three years for 50 Gy *vs.* 55 Gy were (46.7 % *vs.* 81.7 %; p = 0.049) and (52.0 % *vs. *61.9 %; p = 0.842), respectively. The higher BMI levels among the 50 Gy group contributed to the worse outcomes compared to the 55 Gy (43.2% *vs*. 74.1%; p=0.637). T1 tumors treated with > 65 Gy have shown a reduced risk of recurrence compared to T2 and T3 tumors or doses ≤ 65 Gy [28]. Corresponding findings were established in our study, albeit at the lower dose comparison of 50 and 55 Gy; T1 and T2 tumors treated with 55 Gy had significantly increased three-year LC compared to those treated to 50 Gy (p = 0.018). A study by Kaskowitz et al. suggested that Stage I NSCLC patients treated with a median dose of 63.2 Gy showed improved cause-specific survival [29]. Our study demonstrates that, for Stage I NSCLC patients treated with 55 Gy *vs.* 50 Gy, improved OS at three years (86.1 % *vs*. 46.2 %; p = 0.056). The National Comprehensive Cancer Network (NCCN) and American College of Radiology (ACR) have recommended the delivery of BED > 100 Gy_10_ for SBRT [30,31]. Our data shows that BED doses > 100 Gy; 11 Gy x five fractions (n = 38; 33.3 %) had improved three-year OS and LC rates [(81.7 % *vs.* 46.7 %; p = 0.049), (96.6 % *vs.* 66.3 %; p = 0.002)], compared to our previous standard dose of 10 Gy x five fractions (n =76; 66.7 %).

Onimoru et al. conducted a dose-escalation study to determine the optimal SBRT dose for T2 N0 M0 NSCLC and recommended a dose of 50 Gy in four fractions for peripheral tumors with PTV ⩾ 100 cm^3^ [32]. In other studies from Western countries, 54 Gy in three fractions with the dose covering 95 % of the PTV or to the 80 % isodose line has been used for peripheral tumors, while slightly lower doses were prescribed for centrally located tumors, T2 tumors, and tumors with chest wall invasion. All of these studies have reported acceptable toxicities and favorable outcomes [12,14].

In our analysis, we found similar findings of favorable survival outcomes by PTV volume in 55 Gy compared to 50 Gy (91.7 % *vs. *38.3 %; p = 0.049) respectively. In attempting dose escalation, however, the relatively higher incidence of Grade ≥ 2-radiation pneumonitis may be a barrier. Thus, the development of techniques to predict the risk of radiation pneumonitis after SBRT for NSCLC is urgently needed [33-37].

In their second study, Miyakawa et al. reported that dose escalation dose did not lead to improved outcomes, although LC, OS, and toxicity tended to be higher compared to their initial study [38]. Using the National Cancer Data Base (NCDB), Koshy et al. have reported that Stage I NSCLC patients receiving high doses (>150 Gy BED) had significant survival benefit in those patients with T2 tumors [39]. Our study also reports an increased survival benefit in Stage I NSCLC patients (86.1 % *vs.* 46.2 %; p=0.056) along with significant increase in LC of 96.6 % versus 66.3 % for those treated with 55 Gy *vs.* 50 Gy. Post-treatment complications were mild with shortness of breath or cough (6.1 %), scarring (7.0 %) and rib fractures (4.4 %).

In order to treat patients safely with highly conformal, large dose fractions, treatment centers have to have stringent quality control measures in place because inaccurate targeting and treatment of lung lesions can lead to significant morbidity and mortality. In addition, SBRT clinical trials are still necessary to define dose-volume criteria clearly and to establish a role for dosimetry audits or independent plan review in the quality assurance process. Multi-center clinical trials are therefore essential to provide the ideal study power and reduce the likelihood of important outcome and toxicity findings being obscured [40].

Interestingly, for the 55 Gy cohort, the one-, two-, and three-year OS rates are very similar. We hypothesize that if patients survive the first year, they are likely to survive later on. We further hypothesize that similar to head and neck squamous cell carcinoma, NSCLC patients are also likely to fail locally and regionally more in the first year if the doses are inadequate and such local-regional failure can lead to distant metastases later on thus leading to worsening survival.

Limitations

The retrospective nature of this study poses a potential selection bias. The advanced age of this cohort (median of 68 years) also adds to the relatively high competing risk of their death from preexisting medical comorbidities. Additional limitations include the unknown skin and lung toxicity levels in some of this group of patients (22 %), which could have limited the identification of any dose-response relationship.

## Conclusions

The SBRT experience at our institution demonstrates that OS and DFS were significantly better and the likelihood of local, regional, and distant recurrence were lower among early-stage NSCLC patients treated with an SBRT dose of 55 Gy compared to 50 Gy. Our data suggest that an increase in prescribed dose may provide superior OS and LC rates in these patients. However, it is possible that the shorter median follow-up for the 55 Gy cohort compared to the 50 Gy cohort and/or the BMI variations between the two groups might have confounded our results and only a careful continued follow-up and subsequent reporting of our findings are likely to clarify this bias. Additional prospective studies are necessary to confirm these findings and realize an optimal dose fractionation scheme as a function of tumor volume.
